# The uncommon diagnosis of hiatal hernia associated pancreatitis: A case report

**DOI:** 10.1016/j.ijscr.2022.107328

**Published:** 2022-06-20

**Authors:** Atef Mejri, Khaoula Arfaoui, Jasser Yaacoubi, Mohamed Firas Ayadi

**Affiliations:** Department of General Surgery, Jendouba Hospital, Tunisia; Faculty of Medicine of Tunis, Tunis El Manar University, Tunis, Tunisia

**Keywords:** Hiatal hernia, Pancreatitis, Para-esophageal hernia, Pancreatic herniation, Intrathoracic pancreas

## Abstract

**Introduction and importance:**

Hiatal hernia (HH) contents commonly include stomach, transverse colon, small intestine, and spleen but herniation of the pancreas is an extremely rare phenomenon, even rarer when HH is associated with acute pancreatitis.

**Case presentation:**

A 56-year-old female with hypertension and gastroesophageal reflux disease presented with abdominal pain, vomiting and chest discomfort evolving for 24 h. Physical examination revealed left-upper quadrant tenderness without guarding. Blood tests showed elevated serum amylase and lipase levels.

An abdominal CT scan demonstrated a large type-IV hiatal hernia involving the entire stomach, transverse and right colon, small intestine, duodenum as well as the head, body and the tail of pancreas. The pancreas was enlarged consistent with pancreatitis. Patient clinical status improved with conservative treatment.

**Clinical discussion:**

The stomach is the most common organ to herniate through the diaphragm and pancreatic herniation is extremely rare with only few cases in the literature. Even rarer when associated with acute pancreatitis. This diagnosis is a major diagnostic and therapeutic challenge that has to be evoked in elderly presenting with chest pain and a negative cardiopulmonary evaluation. The ideal treatment is still unclear, however, conservative treatment is the initial management and surgery may be considered in case of recurrent episodes of acute pancreatitis.

**Conclusion:**

HH associated with acute pancreatitis is a major diagnostic and therapeutic challenge. Clinicians should consider this rare diagnosis in every case of chest pain with negative cardiopulmonary evaluation.

## Highlights

• Intrathoracic herniation of the pancreas is extremely rare.
• Clinicians should be mindful of its resulting complications.
• Type-IV hiatal hernia may explain certain cases of idiopathic pancreatitis.
• The initial management of acute pancreatitis with intrathoracic pancreas is conservative.

## 1 Introduction

Hiatal hernia (HH) is defined by the permanent or intermittent prolapse of any abdominal organ into the chest through the diaphragmatic esophageal hiatus [1]. Prolapse of the stomach, intestine, transverse colon, and spleen is relatively common. However, large hiatal hernia including the pancreas is extremely rare [2] with only few cases previously described in the literature [3]. Acute pancreatitis secondary to this phenomenon is particularly unusual, as only 7 such cases have been reported [3].

We present here an uncommon case of an overtly symptomatic patient with acute pancreatitis who had herniation of pancreas into the chest.

This case report has been reported in line with the SCARE Criteria 2020 [4].

## 2 Presentation of case

A 65-year-old female with a past medical history of hypertension and gastroesophageal reflux disease symptomatically managed with pantoprazole 40 mg daily, presented to emergency department with one day of abdominal pain and vomiting. Her abdominal pain was located in the left upper quadrant. She described the pain as moderate in intensity, of sudden onset and associated with chest discomfort and dyspnea. She had no significant family history and denied any alcohol abuse. Physical examination showed a heart rate of 82 beats/min, a blood pressure of 147/82 mmHg, a body temperature of 37.4 °C, and a respiratory rate of 22/min. Abdominal examination demonstrated left-upper quadrant tenderness without guarding, the bowel sounds could be perceived on the left chest. Cardiovascular, pulmonary and neurological examination was unremarkable.

Blood examination results showed elevated serum amylase level at 920 U/L, elevated serum lipase level at 1027.85 UI/L, leukocytosis at 15,300 U/L with 84 % neutrophils.

The alanine aminotransferase level was at 367.4 U/L, the aspartate transaminase level at 233.2 U/L, total bilirubin level at 87.41 umol/l and direct bilirubin level at 47.6 umol/l, alkaline phosphatase level at 236 IU/L, and gamma-glutamyl transpeptidase level at 74 IU/L. The renal function was normal.

**Fig. 1:**
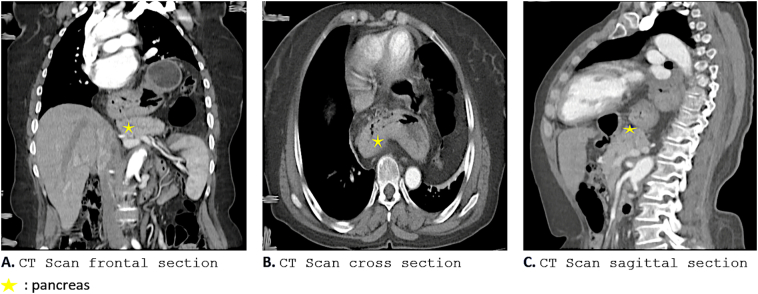
CT scan views showing the Intrathoracic position of the pancreas (yellow asterisk). (For interpretation of the references to colour in this figure legend, the reader is referred to the web version of this article.)

A CT scan of the abdomen demonstrated a large type IV hiatal hernia through the esophageal hiatus. The hernia involved the entire stomach, transverse and right colon, small intestine, duodenum as well as the head, body and the tail of pancreas (Fig. 1). The pancreas was enlarged consistent with acute pancreatitis.

Given her clinical presentation, CT of the abdomen and serum lipase elevation, a diagnosis of mild acute pancreatitis was made and the patient was treated with intravenous hydration with lactated Ringer's Solution, analgesics along with intensive care monitoring. Etiology investigation of pancreatitis was negative arguing in favor of the hernia as a cause of acute pancreatitis in this case.

An esophagogastroduodenoscopy confirmed hiatal hernia without obstruction and excluded the presence of esophagitis. Biopsies revealed chronic gastritis in the antrum of the stomach. We had to respect the declared intention of the patient for a conservative procedure. She was discharged 5 days later. The medical follow up visit two weeks later showed a recovered patient. Upon discussion, we explained that a delayed surgery should be attempted to prevent the increased morbidity and mortality related to a potential recurrent pancreatitis. However, the patient insisted on refusing any surgical intervention.

## 3 Discussion

Hiatal hernias are a part of diaphragmatic hernias [5] and are characterized by the trans-hiatal shifting of the abdominal contents into the chest [2]. The stomach is the most common organ to herniate, other less common herniations include transverse colon, small intestine, and spleen [6].

Because the head segment of the pancreas and duodenum are positioned in the retroperitoneum and held by Treitz's ligament, pancreatic herniation is extremely rare [2], [5]. But stretching of the transverse mesocolon due to increase in intra-abdominal pressure causes loosening of the posterior fascia resulting in pancreatic mobilization and herniation [7]. Pancreatic herniation is a rare occurrence, a review of the literature conducted in 2020, found 17 published cases of intrathoracic herniation of the pancreas [8], [9], with 12 cases occurring in or after the sixth decade of life, and with equal proportion of men and women [3].

As a result, pancreatic hiatal hernia associated with acute pancreatitis is extremely unusual, having been recorded in only 7 cases previously [3]. Though the mechanism of hernia-associated pancreatitis is no not completely elucidated yet, the authors attempted to discuss some possible causes: the parenchymal trauma resulting from repetitive transhiatal protrusion of the pancreas, intermittent stretching and traction of blood vessels such as the vascular pedicle, leading to ischemia lesions, volvulus or intermittent twisting of the pancreatic duct leading to pancreatic secretion against a permenant obstruction [3], [5], [7]. Incarceration of the pancreas resulting in anoxic injuries may also be another mechanism [10].

Symptoms of acute pancreatitis caused by the herniation of pancreas in HH consist primarily of chest and epigastric pain [1], [3]. Other manifestations include vomiting, dyspepsia, dyspnea, and diaphoresis [1], [5]. The diagnosis is confirmed by elevation of pancreatic enzymes and imaging evidence of pancreatic herniation with inflammatory changes suggestive of pancreatitis (peripancreatic fluid, mesenteric stranding).

Cases of pancreatic herniation with pancreatitis are rare; Therefore, the ideal treatment is still unclear [1], [3], [6]. However, conservative treatment including intravenous fluid supplementation, analgesia and early feeding is usually indicated [1], [11], [12].

Historically, HH cases with pancreatitis were treated with surgery [1], [6], [7], [13], [14]. However, the benefit of such intervention remains unknown especially that the rate of recurrence appears to be low [3], [12]. Moreover, the decision to pursue surgical repair should be considered on a case-by-case basis since most affected patients are of advanced age. Thus, for pancreatic herniation, surgical repair may be considered in case of recurrent episodes of acute pancreatitis [15], [16] and a multidisciplinary approach involving a general surgeon, an intensivist, and a cardiothoracic surgeon is recommended [5].

## 4 Conclusion

HH-associated acute pancreatitis represents a serious diagnostic and therapeutic challenge. Although rare, this diagnosis should be considered in patients with inexplicable pain associated with a large HH, or, in elderly presenting with chest pain and a negative cardiopulmonary evaluation. Conservative treatment is usually indicated and a case-by-case evaluation is the most appropriate management approach.

## Abbreviations

HH: hiatal hernia
CT: computerized tomography

## Sources of funding

None.

## Ethical approval

An ethical approval was obtained from the Jendouba Regional Hospital Medical Ethics Committee N° CC19Y22.

## Consent

Written informed consent was obtained from the patient for publication of this case report and accompanying images. A copy of the written consent is available for review by the Editor-in-Chief of this journal on request.

## Authors' contributions

Conceptualization: AM.

Data curation: KA.

Supervision and performing surgery: AM.

Writing - original draft: MFA, JY.

Writing - review & editing: AM.

The final version of manuscript was read and approved by all authors.

## Research registration

Not applicable.

## Guarantor

Atef Mejri.

## Provenance and peer review

Not commissioned, externally peer-reviewed.

## Declaration of competing interest

The authors declare that they have no conflicts of interest.
